# Efficient Removal of Fluoride from Phosphogypsum Leachate: Insights into Microscopic Mechanism of Polyferric Sulfate-Assisted Precipitation via Molecular Simulation

**DOI:** 10.3390/molecules31193524

**Published:** 2026-10-03

**Authors:** Wen Fang, Zhiguo Zhang, Yuefei Zhang, Jin Lv, Nan Yang, Ruan Chi

**Affiliations:** 1 Engineering Research Center of Phosphorus Resources Development and Utilization of Ministry of Education, Wuhan Institute of Technology, Wuhan 430205, China; 2 School of Optoelectronic Materials & Technology, Jianghan University, Wuhan 430056, China; 3 China Wuhuan Engineering Corporation Ltd., Wuhan 430072, China; 4 School of Chemistry and Environmental Engineering, Wuhan Institute of Technology, Wuhan 430205, China

**Keywords:** coagulation, phosphogypsum, fluoride, polyferric sulfate, molecular dynamics

## Abstract

Fine calcium fluoride (CaF_2_) particles formed during fluoride removal processes often exhibit poor aggregation and separation behavior, especially in phosphogypsum (PG) leachate with high soluble fluoride concentrations. In this study, polyferric sulfate (PFS) was employed as a coagulation aid following CaO precipitation to enhance CaF_2_ aggregation and solid–liquid separation, with particular focus on the underlying molecular mechanisms. Experimental results showed that the sequential addition of CaO followed by PFS significantly improved fluoride removal compared with CaO precipitation alone. Under optimal conditions, the sequential CaO/PFS addition achieved a maximum fluoride removal efficiency of 94.1%, compared with 88.3% for the CaO-only system. Molecular dynamics simulations revealed that Fe-containing species promoted CaF_2_ aggregation through electrostatic attraction and bridging interactions involving OH^−^, Ca^2+^, and F^−^. Quantitative analyses based on radial distribution functions, coordination numbers, solubility parameters, and self-diffusion coefficients further demonstrated that pH, ion concentration, and dosing sequence strongly influenced aggregate formation, with sequential dosing showing superior performance over simultaneous addition. This work provides molecular-level insight into inorganic coagulation-assisted CaF_2_ aggregation and offers theoretical support and practical guidance for efficient fluoride removal and resource-oriented treatment of PG leachate.

## 1. Introduction

Phosphogypsum (PG) is a by-product of the wet process of phosphoric acid [1]. Currently, PG is mainly disposed of in landfills and open piles, and the amount of PG piled in China has exceeded 800 million tonnes [2,3]. Soluble fluoride is the harmful impurity in PG [4,5], and when its mass fraction exceeds 0.3%, it will coarsen the crystals of PG hydration products, weaken the molecular forces between crystals, loosen the structure, and reduce the strength of PG [6]. Excessive intake of fluoride in the body may lead to undesirable diseases such as dental fluorosis, osteofluorosis and thyroid disorders [7]. According to the standards set by WHO, the level of fluoride in drinking water should not exceed 1.5 mg/L [8]. Therefore, effective treatment of soluble fluoride in PG is of great significance in reducing human health risks and preventing environmental pollution [9].

Various technologies, including adsorption/ion exchange [10], membrane separation [11], electrochemical treatment [12], reverse osmosis [13,14], and calcium-based precipitation, have been investigated for fluoride removal [15]. Although adsorption and membrane-based processes can achieve effective fluoride removal, their application to high-fluoride wastewater may be limited by adsorbent regeneration, membrane fouling, concentrated waste streams, and relatively high operating costs. In contrast, Ca-based precipitation is particularly suitable for high-fluoride streams because of its low reagent cost and the potential conversion of dissolved fluoride into recoverable CaF_2_ solids [16,17]. However, the fine CaF_2_ particles formed during precipitation often exhibit poor aggregation and settling behavior, which limits efficient solid–liquid separation and subsequent resource recovery [18].

Adding a flocculant promotes the aggregation of suspended particles into larger flocs, which can then be more easily settled or filtered [19,20]. In recent years, significant advances have been made in understanding coagulation or flocculation mechanisms. For instance, it has been shown that C=N bonding enhances interaction and charge neutralization between chitosan-based flocculants and azo dyes, thereby promoting floc formation, as evidenced by comparisons of floc size and zeta potential [21]. The microblock structure of anionic polyacrylamide enhances bridging adsorption and charge neutralization, improving coagulation performance, as demonstrated in turbidity and zeta potential studies [22]. Additionally, studies have shown that a composite flocculant of polymeric ferric chloride and polydimethyl-diallylammonium chloride destabilized the colloidal particles through charge neutralization, double-layer compression, and bridging effects, leading to floc formation and separation from water [20]. However, most of the existing studies on coagulation mechanisms focus on macroscopic physical parameters, with limited investigation into the evolution of microstructural processes.

Molecular Dynamics (MD), as a simulation method based on Newtonian mechanics capable of examining substance interactions and motion behaviors at the molecular scale [23], has advanced significantly in the study of coagulation and flocculation mechanisms in recent years [24,25]. For instance, when examining the flocculation mechanism of quartz and kaolinite with polyacrylamide (HPAM) in brine, molecular dynamics simulations revealed that the flocculation is primarily driven by interactions between deprotonated oxygen in the polymer’s acrylic acid groups and hydroxides on the hydromagnesite surface, along with hydrogen-bonding interactions between nitrogen in the acrylamide groups and hydroxides [26]. Additionally, molecular dynamics simulations have been employed to investigate the mechanism of graphene oxide (GO) as a flocculant for the removal of methylene blue (MB) from water, revealing that the interaction between GO and MB molecules is primarily driven by electrostatic attraction [27].

Polyferric sulfate (PFS), as an inorganic polymeric coagulant, has been widely applied in wastewater treatment due to its strong charge neutralization ability, adsorption capacity, and bridging effect [28]. Previous studies have demonstrated that PFS can effectively remove suspended solids, organic pollutants, phosphorus, and other contaminants from aqueous systems by forming hydrolyzed Fe species and polymeric iron hydroxyl complexes, which promote particle destabilization and aggregation. In particular, the multivalent Fe species generated from PFS hydrolysis can interact with negatively charged colloids through electrostatic attraction, surface adsorption, and hydroxyl-bridging effects, resulting in the formation of larger and denser flocs with improved settling performance [29]. However, previous studies have mainly focused on PFS-based pollutant removal or coagulation of suspended particles, while its role in promoting the aggregation and separation of inorganic precipitates remains unclear. Although calcium-based precipitation is an economical approach for fluoride removal, the resulting fine CaF_2_ particles often exhibit poor aggregation and separation. Therefore, the effects of PFS on CaF_2_ aggregation and the underlying molecular mechanisms require further investigation.

Therefore, this study developed a CaO–PFS precipitation–coagulation strategy for high-fluoride phosphogypsum leachate, with particular emphasis on CaF_2_ aggregation and solid–liquid separation. The effects of CaO/PFS addition sequence on fluoride removal and CaF_2_ aggregation were systematically evaluated through fluoride removal efficiency, zeta potential, particle-size distribution, SEM, and XRD analyses. Molecular dynamics simulations were further employed to elucidate the interactions among Ca^2+^, F^−^, Fe-containing species, SO_4_^2−^, and OH^−^ and to clarify the molecular origin of the experimentally observed sequence effect. The novelty of this work lies in linking sequence-controlled precipitation–coagulation with enhanced CaF_2_ aggregation and separability and providing a molecular-level interpretation of this process. This study therefore provides a mechanistic basis for improving fluoride removal and CaF_2_ resource recovery from high-fluoride phosphogypsum leachate.

## 2. Materials and Methods

### 2.1. Materials

The PG was supplied by Hubei Xingfa Chemicals Group Co., Ltd. (Yichang, China); Analytical-grade CaO, NaF, NaOH, Fe_2_(SO_4_)_3_, citric acid, and trisodium citrate were obtained from National Pharmaceutical Group Chemical Reagent Co., Ltd. (Shanghai, China).

### 2.2. Experimental Methods

#### 2.2.1. Preparation and Characterization of Polyferric Sulfate (PFS)

Polyferric sulfate (PFS) solution was synthesized by dissolving 13.30 g of Fe_2_(SO_4_)_3_ in 40 mL of distilled water under continuous stirring at room temperature. An aqueous NaOH solution (0.23 g/mL) was then added dropwise until the molar ratio of OH^−^ to Fe^3+^ (basicity, B) reached 0.1; higher basicity was avoided to prevent system destabilization and Fe(OH)_3_ precipitation. After aging for 2 h, a transparent reddish-brown PFS solution (B = 0.1) was obtained and used as the coagulant in subsequent experiments [30]. The prepared PFS solution was characterized using a Thermo Fisher DXR2i micro-Raman spectrometer (Thermo Fisher Scientific, Waltham, MA, USA) with a laser wavelength of 785 nm over a wavenumber range of 50–4000 cm^−1^.

#### 2.2.2. Removal of Fluoride

We mixed 20.0 g PG with water at a solid–liquid mass ratio of 1:10 and stirred at 500 rpm for 40 min. The mixture was filtered to obtain a PG leachate, which was stored for further use. At 25 °C, 40 mL of this leachate was measured, and 20 mg of CaO was added, followed by stirring at 300 rpm for 30 min. After the CaO reaction, 11 mg of PFS was introduced, and the reaction was stirred at 100 rpm for an additional 20 min before filtering the supernatant. The residual fluoride content was determined using an ion-selective electrode (ISE), with citric acid and trisodium citrate used to prepare a total ionic strength-adjusting buffer solution (pH = 5). The pH of the supernatant was measured with a pH electrode. All experiments were conducted in triplicate (n = 3), and the results are presented as mean values with standard deviations (mean ± SD).

#### 2.2.3. Characteristics of the Precipitation

The two precipitates obtained by adding only calcium oxide and coagulation with PFS were characterized by XRD (Bruker D8 ADVANCE, Bruker AXS GmbH, Karlsruhe, Germany) and SEM (Thermo Scientific Quattro S, Thermo Fisher Scientific, Waltham, MA, USA). After the precipitation experiments, the solid products were collected by filtration, thoroughly washed with deionized water to remove residual soluble ions, and then dried at 60 °C for 24 h. The dried precipitates were subsequently stored in a desiccator prior to SEM and XRD analysis to avoid moisture adsorption.

Fluoride ion concentrations were determined using a fluoride ion-selective electrode (PF-202, Shanghai INESA Scientific Instrument Co., Ltd. (LeiCi), Shanghai, China). Prior to each set of measurements, the electrode was calibrated daily with a series of NaF standard solutions (10^−5^–10^−1^ mol/L) prepared in a Total Ionic Strength Adjustment Buffer (TISAB) to maintain constant ionic strength and minimize interference. A calibration curve of electrode potential versus pF(−lg[F^−^]) was established, showing a linear Nernstian response with a correlation coefficient (R^2^) greater than 0.999. Solution pH was monitored and adjusted using a digital pH meter (E-301, Shanghai INESA Scientific Instrument Co., Ltd. (LeiCi), Shanghai, China). The pH electrode was calibrated before each use with standard buffer solutions at pH 4.01, 6.86, and 9.21 to ensure measurement accuracy.

### 2.3. MD Simulation Details

#### 2.3.1. Model Construction

To investigate the PFS-assisted CaF_2_ precipitation at the microscopic level, a cubic water box with a side length of 7 nm containing Ca^2+^ and F^−^ (fluoride concentration of 2.8 g/L) was constructed to simulate the aggregation and precipitation of Ca^2+^ and F^−^. The pH of the simulation system was controlled by adjusting the number of OH^−^ ions in the water box. Based on the ionization equilibrium of water and the volume of the simulation box, 0, 2, 20, and 200 OH^−^ ions were introduced to represent pH 7, 12, 13, and 14, respectively. The corresponding numbers of OH^−^ ions for each simulation system are summarized in Table 1. These systems were designated as FCS-1, FC-2, FC-3, and FC-4, respectively. Since Ca^2+^ was present in excess under the experimental conditions, additional Ca^2+^ ions were introduced as necessary to maintain charge neutrality in the simulation systems.

Polymerized ferric sulfate (PFS) is an inorganic polymer coagulant with the general formula [Fe_2_(OH)_n_(SO_4_)_3−n/2_]_m_, where m is the degree of polymerization and n is less than 2 [28]. In aqueous solution, PFS consists of a mixture of monomeric, hydrolyzed, and polymeric ferric species that exhibit complex and dynamic structural configurations. Explicitly representing these polymeric clusters in molecular dynamics simulations would require substantially increased computational resources, extensive parameterization, and potentially the use of reactive force fields, which are beyond the scope of the present study. Therefore, Fe^3+^ and SO_4_^2−^ ions were employed as representative species of PFS to simplify the simulation model. By simulating their interactions with Ca^2+^ and F^−^ under corresponding pH conditions, the dominant mechanisms, including electrostatic interactions and ionic coordination, can be effectively captured while reducing computational complexity. Ca^2+^, F^−^, Fe^3+^, and SO_4_^2−^ were added to simulate the precipitation and aggregation of calcium ions and fluoride ions in solution when CaO and PFS were added simultaneously to fluoridated wastewater (PFS concentration 2.75 g/L) (named the FCS-1 system in Table 1). In the FCS-2 system, Ca^2+^ and F^−^ were introduced initially, followed by the addition of Fe^3+^ and SO_4_^2−^ after 40 ns to simulate a process where CaO is added first, with PFS coagulation subsequently assisting in the precipitation of CaF_2_. Table 1 presents the particle counts for the different systems.

#### 2.3.2. Simulation Parameters

The force field used in the calculation process is the GAFF (Generalized Amber Force Field) force field [31], the SPC/E (Extended Simple Point Charge) water model was utilized [32]. The structures of OH^−^ and SO_4_^2−^ were optimized by Gaussian 16 software at the B3LYP-D3(BJ)/6-31+G* level using density functional theory (DFT) [33]. The bond length and bond angle parameters of these ions in the GAFF force field were derived by Sobtop software (version 1.0) based on the Hessian matrix calculated by Gaussian; for Fe and Ca, the Universal Force Field (UFF) was employed [34]. RESP (Restrained Electrostatic Potential) charge of ions was calculated by Multiwfn 3.8 (dev) code [35]. To prevent kinetic instability from excessive initial force on certain atoms, the maximum interatomic force was limited to less than 1000 kJ/(mol⋅nm), and the energy minimization of the system was carried out using the conjugate gradient (CG) method [36]. Temperature and pressure were maintained at a constant 298.15 K and 1 bar using the V-rescale thermostat and the Parrinello-Rahman barostat. The NVT equilibration time was set at 100 ps with a 2 fs timestep. After achieving temperature equilibrium, an NPT ensemble was run for 100 ps with a 2 fs timestep. Subsequently, MD simulations were conducted under isothermal and isobaric conditions for various durations with a 2 fs timestep to obtain the final results, and the trajectory of each ion was recorded [37,38]. The leap-frog algorithm was used for dynamics, electrostatic interactions were calculated by the Particle Mesh Ewald (PME) method, and Van der Waals interactions were managed using a truncation method with a cutoff distance of 1 nm [39]. All simulations were conducted using GROMACS 2020.4 software [40]. This version was selected because it provides a stable and validated platform compatible with the force fields and analysis methods used in this study.

#### 2.3.3. Kinetic Evaluation Parameters

Radial distribution function (RDF): A characteristic physical quantity that reflects the structure of the microsystem and represents the ratio of the probability density of the occurrence of another atom at a distance *r* around an atom relative to the randomly distributed probability density, which is defined as:

(1) $$\begin{array}{c} g_{AB}\left(r\right)=\frac{1}{4\pi r^{2}\rho_{AB}dr}\frac{\sum_{t=1}^{k}\sum_{j=1}^{N_{AB}}\Delta N_{AB}\left(r\to r+dr\right)}{N_{AB}\times K} \end{array}$$

where $\rho_{AB}$ is the density of the system, dr is the distance interval width, $\Delta N_{AB}$ is the number of B (or A) atoms appearing in the range r to r+dr from an A (or B) atom, N is the number of A and B atoms in the system, and K is the number of time steps.

Coordination number (CN): It is the cumulative number of the RDF and is the average number of particles within a distance r. It can quantitatively characterize the coordination of the number of other particles around the particle, which is defined as:

(2) $$\begin{array}{c} n_{AB}\left(r\right)=4\pi \rho_{B}\int_{0}^{r}\;r^{2}g_{AB}\left(r\right)dr \end{array}$$

where $g_{AB}$ denotes the radial distribution function; $\rho_{B}$ denotes the number density of atoms [41].

The diffusion coefficient (D): It also called the self-diffusion coefficient, is a physical quantity that describes the process of diffusion of a substance in space, following the slope of Einstein’s formula through the mean-square displacement (MSD), which is defined as:

(3) $$\begin{array}{c} MSD=\;\left\langle |r(t)-r(0){|}^{2}\right\rangle \end{array}$$

(4) $$\begin{array}{c} D=\underset{t\to \infty }{lim}\;\frac{MSD\left(t\right)}{6t} \end{array}$$

where r(0) denotes the position of the particle at the initial moment of the simulation, r(t) denotes the position of the particle at the moment t, and the parentheses denote the average value of the whole system [42].

Solubility Parameter (SP): The solubility parameter (δ) is a thermodynamic descriptor related to the cohesive energy density (CED) of a system and reflects the overall strength of intermolecular interactions. It is defined as the square root of the cohesive energy density. According to the Hansen solubility parameter framework, the total solubility parameter can be decomposed into three contributions arising from dispersion forces (*δ_d_*), polar interactions (*δ_p_*), and hydrogen-bonding interactions (*δ_h_*), expressed as:

(5) $$\begin{array}{c} \delta_{t}^{2}=\delta_{d}^{2}+\delta_{p}^{2}+\delta_{h}^{2} \end{array}$$

where $\delta_{t}^{2}$ is the total solubility parameter in (KJ/m^3^)^0.5^, $\delta_{d}^{2}$ is the dispersion force interaction energy, $\delta_{p}^{2}$ is the polarity interaction energy, and $\delta_{h}^{2}$ is the hydrogen bonding interaction energy [43].

## 3. Results and Discussions

### 3.1. Structural Characterization Analysis of PFS

**Figure 1:**
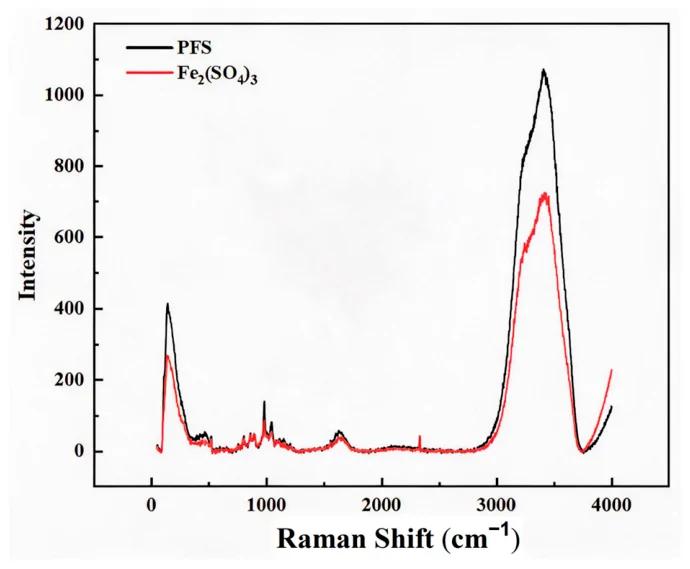
Raman Spectroscopic Characterization of Fe_2_(SO_4_)_3_ and Polyferric Sulfate (PFS) with a Basicity of 0.1.

Figure 1 presents a comparison of the Raman spectra of PFS with a basicity of 0.1 and Fe_2_(SO_4_)_3_. According to the Raman spectroscopic analysis, both PFS and Fe_2_(SO_4_)_3_ exhibit characteristic peaks at 145 cm^−1^ and 3400 cm^−1^, but there are significant differences in peak shape and intensity, reflecting structural differences between the two. At 3400 cm^−1^, PFS shows a markedly stronger O-H stretching vibration peak, primarily attributed to the polynuclear hydroxyl-bridged structures (e.g., Fe–OH–Fe) formed during the polymerization process. This structure not only increases the number of hydroxyl groups but also constructs a three-dimensional network through μ-OH bonds [43]. In contrast, the weak peak of Fe_2_(SO_4_)_3_ at this position arises only from isolated hydroxyl groups in mononuclear hydrated ions or trace oligomers. In the low-frequency region around 145 cm^−1^, the broad peak of PFS corresponds to lattice vibrations of Fe–O–Fe bridging bonds, confirming its polymeric state, whereas the narrower and sharper signal of Fe_2_(SO_4_)_3_ reflects its ionic crystal structure. These structural differences determine their coagulation mechanisms: the abundant hydroxyl-bridged sites in PFS can regulate surface charge via protonation, while its amorphous polymer network enhances its ability to capture colloidal particles. In comparison, Fe_2_(SO_4_)_3_ relies solely on simple charge neutralization by Fe^3+^ hydrolysis products and lacks the spatial bridging effect of polynuclear polymers, resulting in limited coagulation efficiency and application scope. In this study, the PFS coagulant prepared following the procedure described in Section 2.2.1 was used to carry out the subsequent experiments.

### 3.2. Study of Conditions for Coagulation-Assisted Precipitation of Fluoride

**Figure 2:**
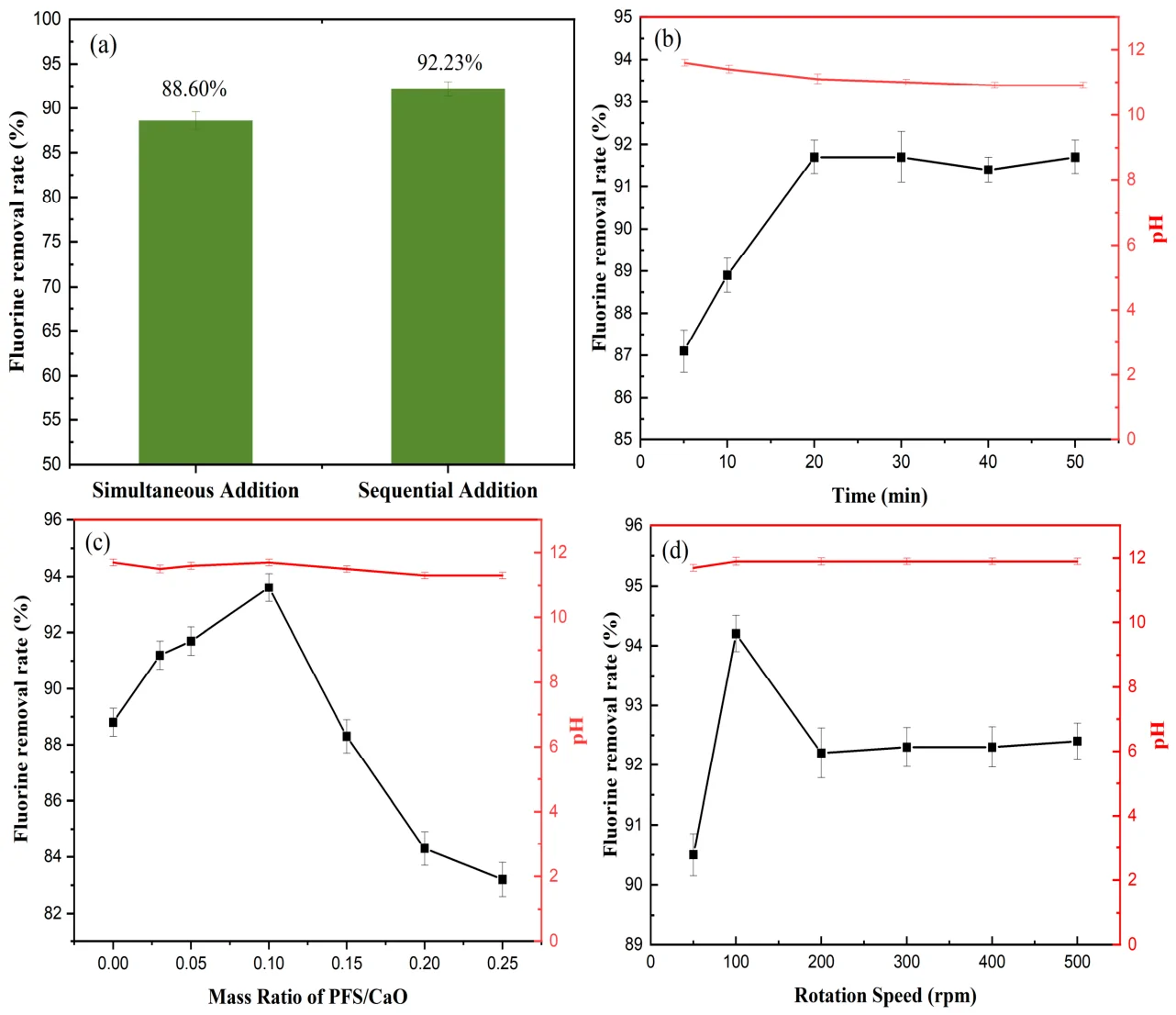
Fluoride removal efficiency and pH of PFS under different (**a**) addition sequences, (**b**) coagulation times, (**c**) dosages, and (**d**) stirring speeds.

All experiments on the precipitation of CaF_2_ were performed in triplicate (n = 3). Error bars representing the SD have been added to the corresponding figures (Figure 2). Statistical significance among multiple experimental conditions was assessed using one-way ANOVA followed by Tukey’s HSD test, with *p* < 0.05 considered statistically significant. The detailed analysis is presented as follows:

The fluoride concentration in the phosphogypsum leachate was 280.48 mg/L. As the CaO dosage increased from 15 mg to 20 mg (per 40 mL solution), the fluoride removal efficiency significantly increased from 75.4% to 91.2%. However, further increasing the CaO dosage beyond 20 mg did not produce a noticeable improvement, and the removal efficiency gradually reached a plateau. This indicates that a CaO dosage of 20 mg provides a sufficient calcium supply to drive CaF_2_ precipitation.

In addition, increasing the reaction time from 10 min to 30 min enhanced the fluoride removal efficiency from 90.8% to 96.4%, after which the system approached equilibrium and further extension of reaction time had a negligible effect. Based on these optimization results, 20 mg CaO and a reaction time of 30 min were selected as the optimal precipitation conditions.

To evaluate the effect of CaO/PFS dosing strategies, two sets of 40 mL leachate samples were prepared. In the first one, 20 mg CaO and 11 mg of PFS were added simultaneously and stirred at 300 rpm for 50 min. In the second, 20 mg CaO was added first (300 rpm, 30 min), followed by 11 mg PFS (100 rpm, 20 min). After the reaction, the supernatant was filtered, and residual fluoride concentration and pH were measured. As shown in Figure 2a, sequential addition achieved higher fluoride removal (92.23 ± 0.80%) than simultaneous addition (88.60 ± 1.00%, *p* = 0.009). This is attributed to the formation of primary CaF_2_ nuclei during the initial CaO stage, which promotes subsequent PFS-induced aggregation. In contrast, simultaneous addition leads to competitive interactions between PFS-derived species (Fe^3+^ and SO_4_^2−^) and F^−^ for Ca^2+^, hindering CaF_2_ nucleation and reducing removal efficiency.

To evaluate the effect of coagulation time on the fluoride removal, six 40 mL leachate samples were treated with 11 mg PFS at 100 rpm for 5–50 min after the precipitation. As shown in Figure 2b, increasing coagulation time from 5 to 20 min improved fluoride removal from 87.2% to 90.9%, after which it plateaued. Meanwhile, pH decreased from 12.2 to 11.3 with time. This trend is attributed to PFS hydrolysis, which generates hydroxyl complexes that enhance charge neutralization and floc formation [44]. Within 20 min, hydrolysis and the coagulation approach equilibrium, leading to stable removal efficiency [45].

To assess the effect of the PFS amount on the fluoride removal and solution pH, seven sets of 40 mL leachate samples were prepared. After CaO precipitation, PFS were added at a PFS/CaO mass ratio of 0–0.25, followed by coagulation at 100 rpm for 20 min. As shown in Figure 2c, increasing the PFS/CaO mass ratio from 0 to 0.1 improved fluoride removal from 88.9% to a maximum of 92.8% (11 mg of PFS), with minimal pH variation. Further increases (>0.1) reduced removal efficiency, dropping to 83.7% at 0.25. This decline is attributed to excessive PFS hydrolysis producing Fe(OH)_3_ colloids that adsorb and block Ca^2+^, hindering its reaction with residual F^−^. Moreover, excess Fe^3+^ introduces charge restabilization, inhibiting particle aggregation and lowering fluoride removal efficiency.

To evaluate the effect of coagulation stirring speed on fluoride removal and pH, six 40 mL leachate samples were prepared. After precipitation, 11 mg PFS was added, and coagulation was performed at 50–500 rpm for 20 min. As shown in Figure 2d, increasing the stirring speed from 50 to 100 rpm enhanced fluoride removal from 90.7% to a maximum of 94.1%. At higher speeds (>100 rpm), the removal efficiency decreased slightly and stabilized at about 92%. This behavior is attributed to insufficient mixing at low speeds and floc breakage due to excessive shear at high speeds. The pH remained stable at about 12, indicating it was mainly governed by PFS dosage and coagulation time.

Under optimal conditions, 20 mg CaO was added to 40 mL fluoride-containing leachate, and they reacted at 300 rpm for 30 min; this is followed by 11 mg PFS and coagulation at 100 rpm for 20 min. This combined process achieved a fluoride removal efficiency of 94.1%, with a residual fluoride concentration of 16.5 mg/L and pH about 12. This value is clearly below the secondary discharge limit of 20 mg/L specified in the Integrated Wastewater Discharge Standard (GB 8978-1996) [46], indicating that the treated effluent meets the basic national discharge requirement. The results also indicate a pronounced change in the surface charge of suspended particles after PFS addition. The average zeta potential of the CaF_2_ suspension in the CaO-only system was approximately −10.90 mV, which increased to about −2.8 mV after adding PFS, approaching the charge-neutralization region and indicating effective destabilization of the colloidal system.

**Figure 3:**
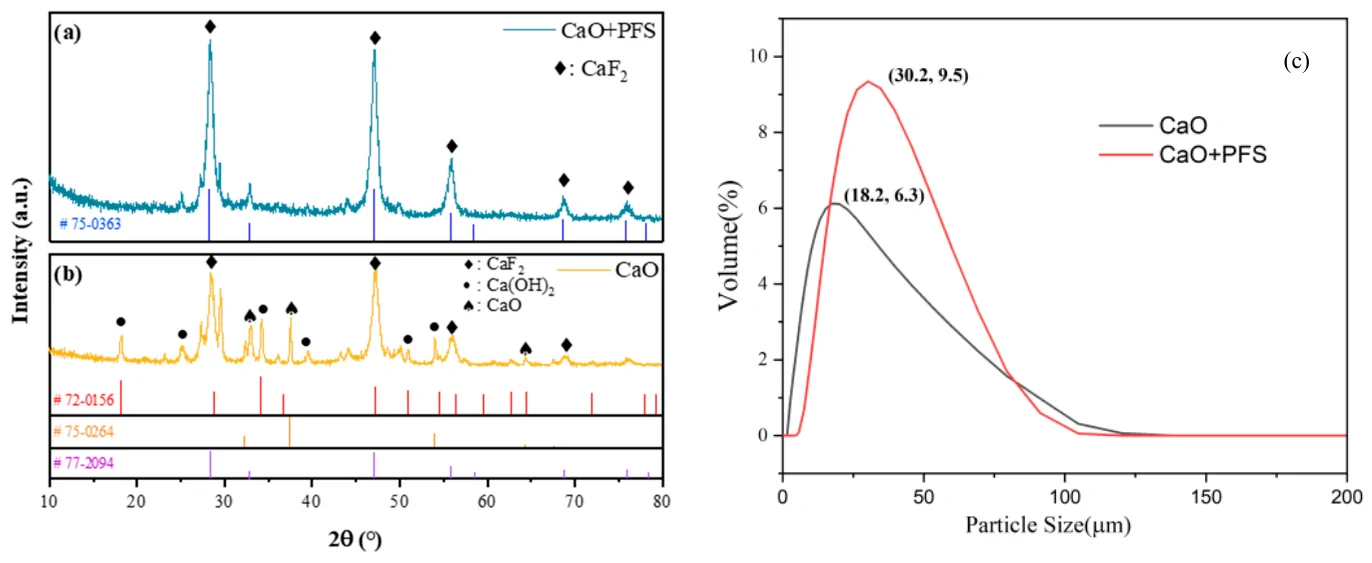
XRD patterns of precipitates and particle size distributions of reaction products: (**a**) CaO and PFS addition; (**b**) CaO addition alone; (**c**) particle size distributions of CaO and CaO–PFS systems. (The symbols “#” indicate the PDF card numbers of the corresponding reference crystalline phases.)

Figure 3c presents the particle size distributions of the CaO and CaO-PFS reaction systems. As shown in the figure, the CaO-only system exhibits a dominant particle size of 18.2 μm with a maximum volume fraction of 6.3%. After the addition of PFS, the main particle-size peak shifts to 30.2 μm, while the maximum volume fraction increases to 9.5%, accompanied by a substantial increase in the overall particle size distribution. These results indicate that PFS effectively promotes the aggregation of fine CaF_2_ precipitates into larger flocs, thereby enhancing sedimentation behavior and improving solid–liquid separation efficiency.

A stoichiometric analysis shows that 40 mL leachate (280.48 mg/L fluoride concentration) contains 11.22 mg (0.59 mmol) F^−^, while 20 mg CaO theoretically removes 13.56 mg F^−^, indicating excess Ca^2+^. However, CaO alone achieved only 75.6% removal (2.74 mg residual), suggesting limitations from CaF_2_ solubility, reaction kinetics, and possible complexation. The addition of PFS enhances CaF_2_ particle aggregation and settling via charge neutralization and bridging, overcoming these limitations and increasing removal efficiency to 94.1%, with residual fluoride reduced to 0.66 mg [47].

### 3.3. Characterization of Precipitates

Under the optimized sedimentation and coagulation conditions described above, two 800 mL leachate samples were prepared; in the first group, 400 mg of CaO was added, and the reaction was conducted at 300 rpm for 30 min. After the precipitation reaction, 220 mg of PFS was added, followed by coagulation at 100 rpm for 20 min, and then the mixture was filtered. In the second group, 400 mg of CaO was added, and the reaction proceeded at 300 rpm for 30 min before filtering. The resulting precipitates from both groups were washed, dried and subsequently analyzed by XRD and SEM characterization.

Figure 3 shows the XRD patterns of the precipitates obtained under different treatment conditions. The characteristic diffraction peaks of CaF_2_ were observed in both groups, with the main peaks corresponding to the (111), (220), and (311) crystal planes, confirming that fluoride removal mainly occurred through CaF_2_ precipitation. Compared with the CaO-only system (Figure 3b), the CaO/PFS sequential addition system (Figure 3a) exhibited stronger CaF_2_ diffraction peaks and weaker peaks associated with residual CaO and Ca(OH)_2_, indicating enhanced conversion of calcium species into crystalline CaF_2_. In the CaO-only system, distinct CaF_2_ peaks were also detected, confirming the effectiveness of CaO-induced fluoride precipitation. However, the relatively stronger CaO and Ca(OH)_2_ peaks suggest that some calcium species remained unreacted. The addition of PFS promoted CaF_2_ aggregation and improved calcium utilization through the coagulation and bridging effects of Fe-containing species, thereby enhancing fluoride precipitation. Several weak peaks may be attributed to minor calcium-containing phases originating from sulfate and phosphate impurities in phosphogypsum leachate. No obvious crystalline Fe-containing phases were detected, suggesting that PFS mainly acted as a coagulation aid rather than forming new crystalline products [48].

**Figure 4:**
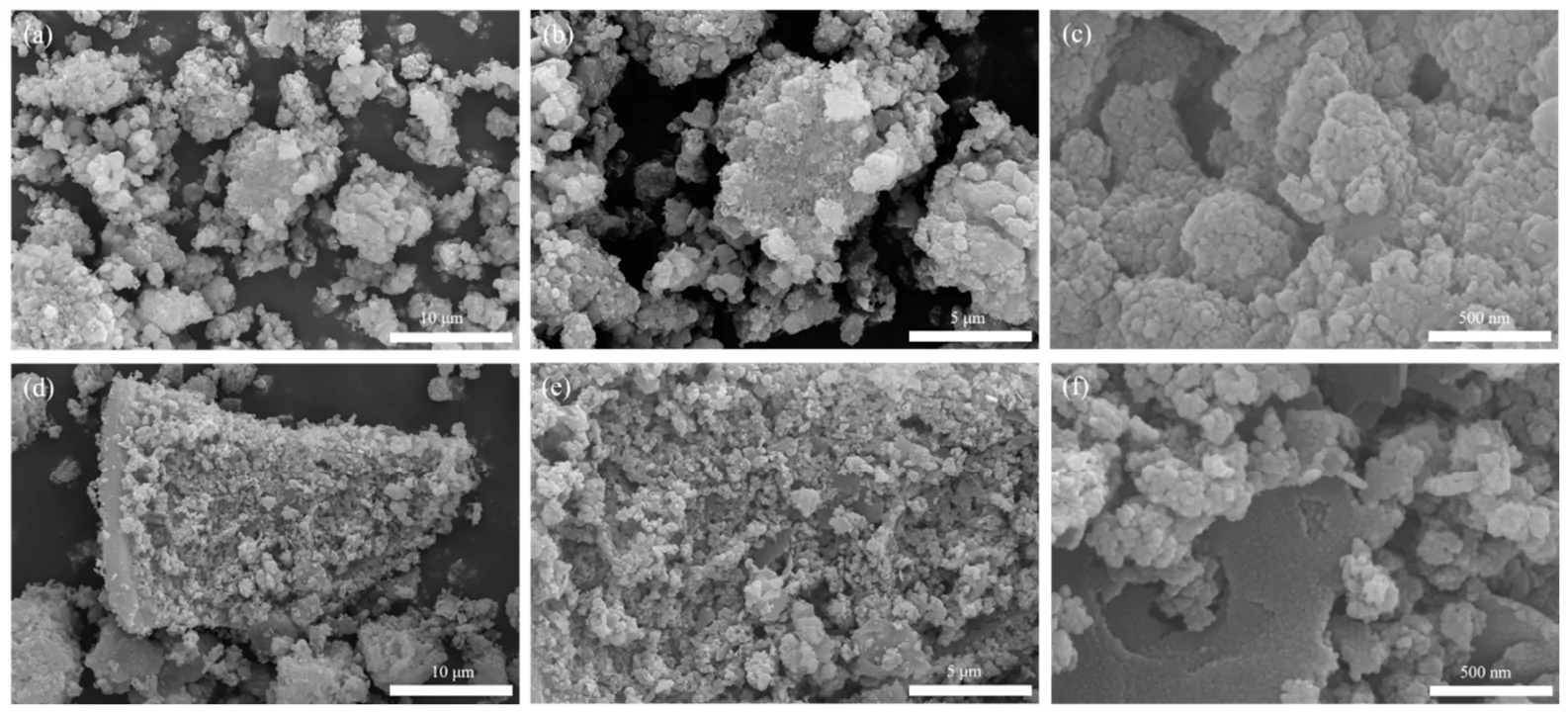
SEM images of precipitation and coagulation under different conditions. (**a**–**c**) CaO addition only. (**d**–**f**) Combined addition of CaO and PFS.

Figure 4 shows the SEM images of the precipitate products under different conditions. At the same magnification, the precipitate in the CaO + PFS group (Figure 4d–f) appeared as larger and denser aggregates, whereas the precipitate in the CaO-only group (Figure 4a–c) consisted of smaller and more dispersed particles. The morphological differences originate from the bridging and agglomeration effects of PFS: the polynuclear hydroxyl-bridged structures formed by Fe^3+^ hydrolysis can connect individual CaF_2_ particles, while SO_4_^2−^ mediates charge neutralization between Ca^2+^ and Fe^3+^, promoting the formation of large and dense aggregates. These larger aggregates exhibit faster settling rates and reduce the adsorption of F^−^ on the surfaces of fine particles, thereby enhancing fluoride removal efficiency.

### 3.4. Calcium Fluoride Aggregation Process Simulated by Molecular Dynamics

#### 3.4.1. Equilibrium Validation of MD Simulations

Before analyzing the molecular interactions and structural characteristics, the equilibrium state of the MD systems was evaluated by monitoring total energy, temperature, density, and RMSD of the FCS-1 system during the 40 ns production trajectory. As shown in Figure S1, the total energy remained stable with only small fluctuations around −1.5 × 10^6^ kJ mol^−1^, indicating that the system maintained a stable energetic state. The temperature fluctuated around 298 K, confirming effective temperature regulation during the simulation. The density remained approximately constant at 1008 kg m^−3^, suggesting that the system reached stable volume conditions under the NPT ensemble. The RMSD increased rapidly during the initial relaxation stage and reached a stable plateau after approximately 7 ns, indicating that the molecular configuration became equilibrated. Therefore, the subsequent structural analyses were performed using equilibrated trajectory data to obtain statistically reliable molecular-level information.

#### 3.4.2. Effect of pH and Structural Evolution of Ca^2+^–F^−^ Association

**Figure 5:**
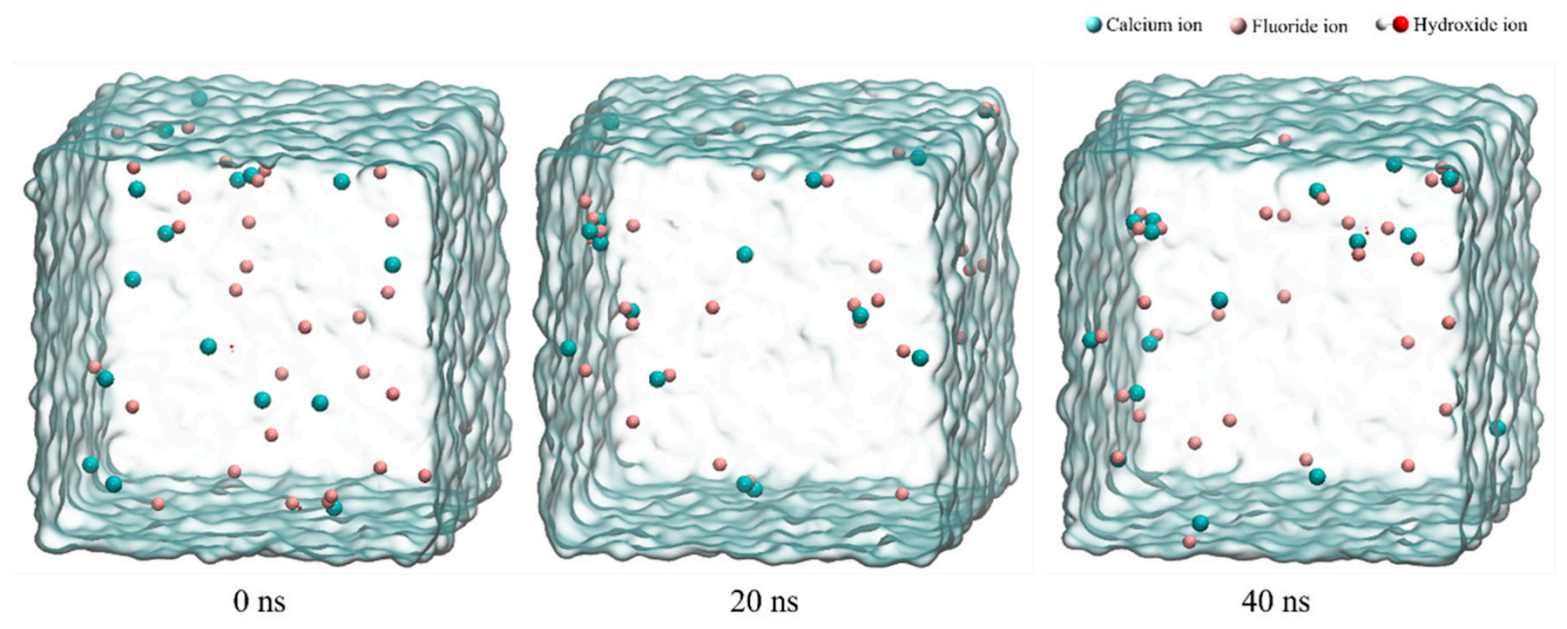
Simulation snapshots of the FC-2 system at different simulation stages.

To examine the structural evolution and stability of Ca^2+^–F^−^ association under different pH conditions, production MD simulations were performed for 40 ns, and representative sampling points at 5, 10, 20, and 40 ns were selected from each production trajectory for analysis. Figure 5 shows representative snapshots of the equilibrated FC-2 system obtained from different trajectory segments, which were used to evaluate the structural characteristics of Ca^2+^–F^−^ association. The radial distribution function (RDF) and cumulative coordination number of Ca^2+^–F^−^ pairs were calculated from the equilibrated trajectory data to characterize their spatial distribution and coordination environment. Comparisons of RDFs and coordination numbers among representative sampling segments were performed to evaluate structural fluctuations within each pH system.

**Figure 6:**
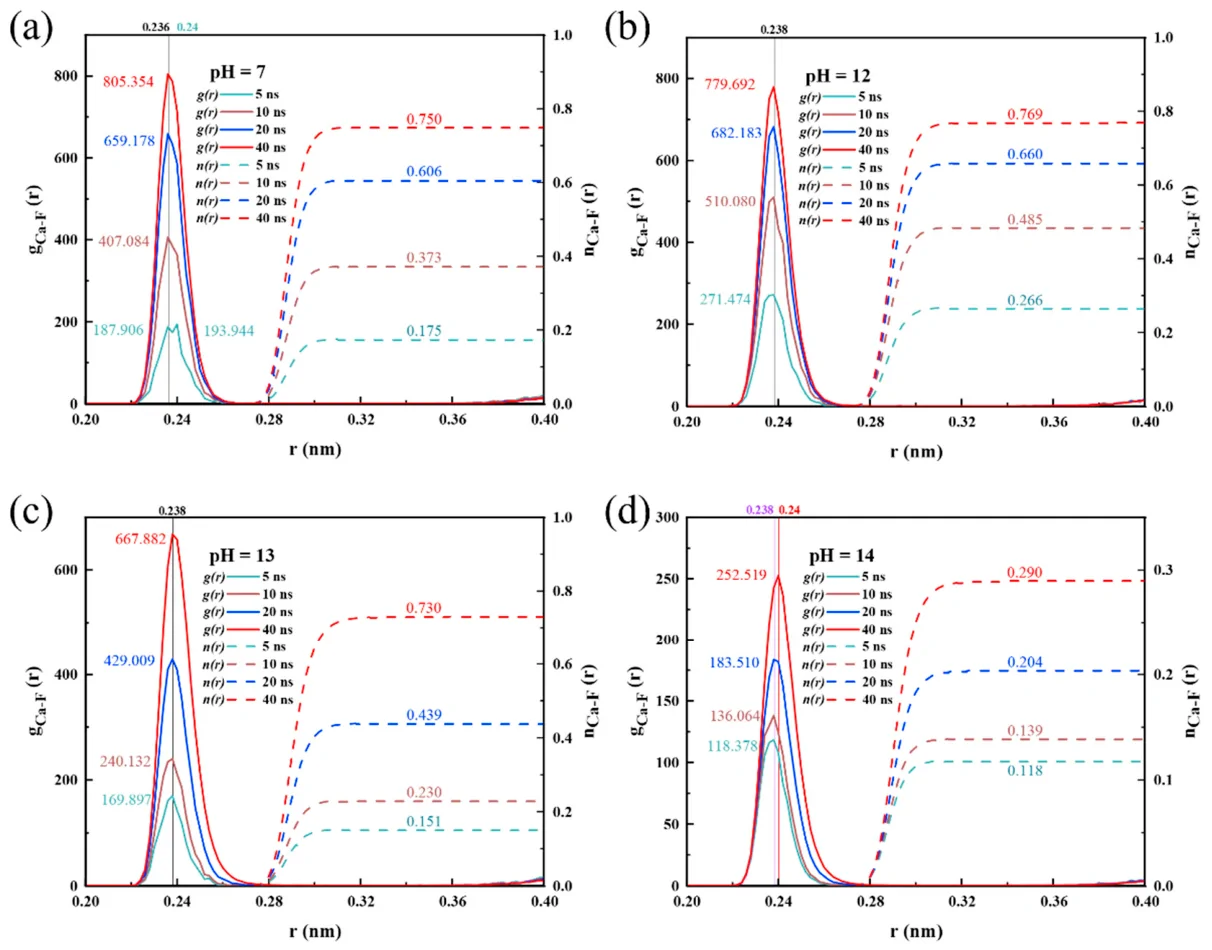
Radial distribution functions g_Ca-F_(r) and coordination numbers n_Ca-F_(r) at different simulation times under various pH conditions with CaO addition alone: (**a**) pH 7; (**b**) pH 12; (**c**) pH 13; and (**d**) pH 14.

In Figure 6, g_Ca-F_(r) represents the RDF of Ca^2+^ and F^−^, while n_Ca-F_(r) denotes their cumulative coordination number. The RDF profiles and coordination numbers calculated from equilibrated trajectories show that Ca^2+^–F^−^ interactions exhibit stable coordination characteristics under different pH conditions. The peak positions remain within 0.236–0.240 nm, indicating similar coordination distances. For example, at pH 7, g_Ca-F_(r) peaked at 193.944 at 5 ns and 805.354 at 40 ns, whereas at pH 14, the peak value of g_Ca-F_(r) was only 252.519 after 40 ns. This difference may be due to the high number of calcium ions used to balance the charge in the FC-4 system. Additionally, n_Ca-F_(r) values consistently increased across different trajectory segments under various pH levels. At pH 14, the FC-4 system had 115 calcium ions, significantly outnumbering F^−^, which explains the lower coordination number of Ca^2+^ with F^−^ at this pH.

To investigate the effect of pH on Ca^2+^-F^−^ aggregation, simulations were performed for 40 ns at various pH levels. The system’s potential and Coulomb interaction energy were extracted from the energy file, and the solubility parameter was calculated as an indicator of particle aggregation [43]. The FC-2 system had the lowest solubility parameter (123.622 (KJ/m^3^)^0.5^), suggesting the strongest intermolecular interactions and the highest aggregation tendency. Among the simulated pH conditions, pH 12 exhibited the most favorable Ca^2+^–F^−^ association and aggregation behavior, thereby facilitating CaF_2_ formation. This pH condition is consistent with the final pH of approximately 12 measured in the experimental precipitation–coagulation system (Figure 2), demonstrating good correspondence between the simulation and experimental conditions.

#### 3.4.3. Impact of CaO and PFS Dosing Sequence

**Figure 7:**
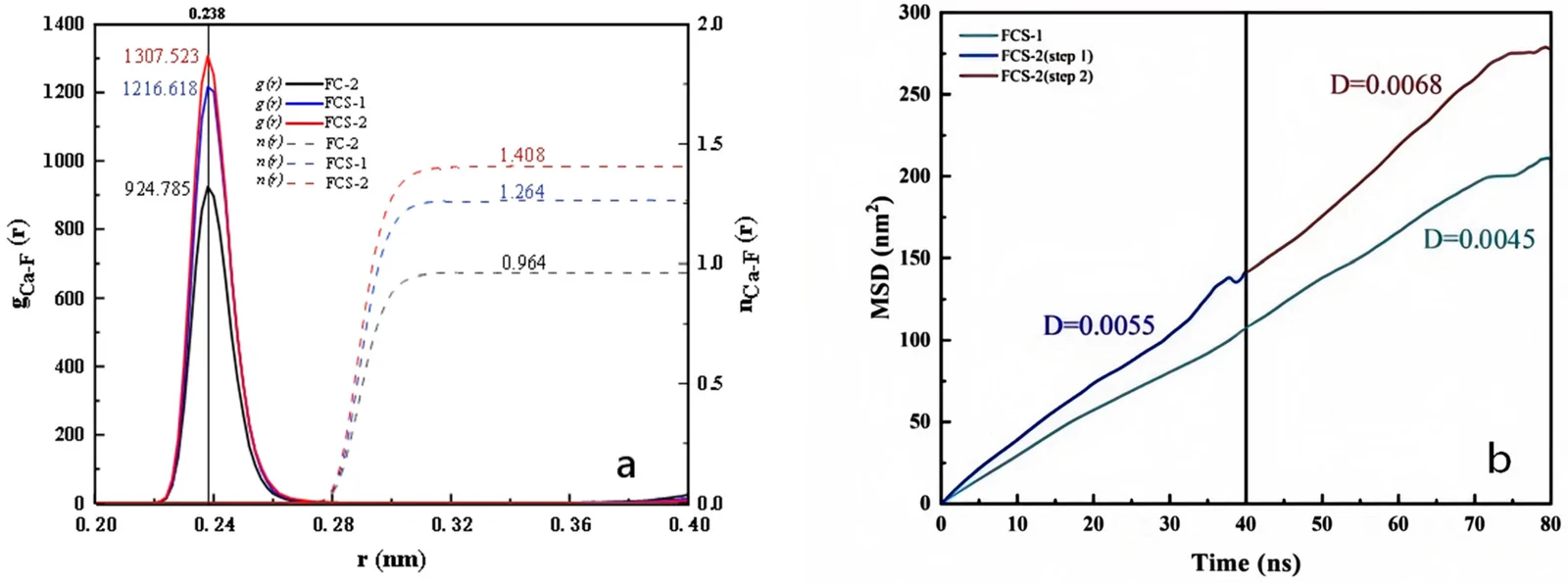
Molecular dynamics simulation results of Ca^2+^–F^−^ interactions and F^−^ diffusion behavior under different CaO/PFS addition strategies: (**a**) Radial distribution functions (g_Ca–F_(r)) and coordination numbers (n_Ca–F_(r)) of Ca^2+^–F^−^ pairs in the FC-2, FCS-1, and FCS-2 systems; (**b**) Mean square displacement (MSD) curves and self-diffusion coefficients of F^−^ ions in the FCS-1 system, FCS-2 system during Step 1 (CaO addition only), and FCS-2 system during Step 2 (subsequent PFS addition).

To investigate the effect of the CaO/PFS addition sequence on the CaF_2_ aggregation, 80 ns production MD simulations were performed for the FC-2, FCS-1 and FCS-2 systems. In the FCS-1 system, Ca^2+^, F^−^, Fe^3+^ and SO_4_^2−^ were introduced simultaneously and simulated for 80 ns. In the FCS-2 system, Ca^2+^ and F^−^ were first simulated for 40 ns, after which Fe^3+^ and SO_4_^2−^ were introduced and the simulation was continued for another 40 ns to represent sequential addition. The Ca-F radial distribution function, g_Ca-F_(r), and the coordination number, n_Ca-F_(r), obtained from the production trajectories were used to characterize Ca^2+^–F^−^ association and evaluate the effect of reagent addition sequence on CaF_2_ aggregation. The results are shown in Figure 7a, where g_Ca-F_(r) in the FCS-2 system peaked at 1307.523 at 0.238 nm, and n_Ca-F_(r) reached 1.408; both of them are larger than the FCS-1 system. The self-diffusion coefficient of F^−^ was further analyzed as a complementary descriptor of ionic mobility [49]. As shown in Figure 7b, the F^−^ diffusion coefficients followed the order D_(FCS-2,step2)_ > D_(FCS-2,step1)_ > D_(FCS-1)_. The higher F^−^ mobility in the FCS-2 system facilitates ion transport and increases the frequency of encounters between Ca^2+^ and F^−^, thereby favoring Ca^2+^–F^−^ association and subsequent CaF_2_ cluster formation and growth. These simulation results are consistent with the experimental findings described above, further demonstrating that the addition of CaO prior to PFS is more favorable for CaF_2_ aggregation.

#### 3.4.4. Structural Evolution of Ca^2+^–F^−^ Association During the Production Trajectory

**Figure 8:**
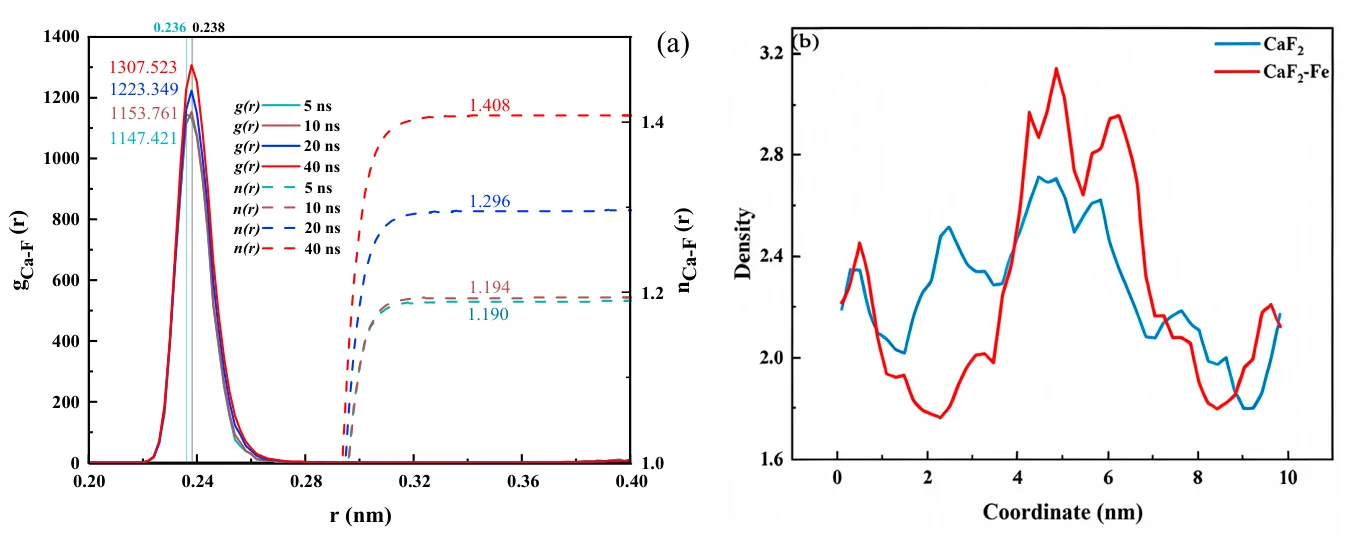
Structural evolution of Ca-F associations along the production trajectory in the FCS-2 system: (**a**) radial distribution functions (g_Ca-F_(r)) and coordination numbers (n_Ca-F_(r)) at representative sampling points; (**b**) spatial density distribution of F^−^.

To examine the structural evolution of Ca^2+^–F^−^ association following the introduction of Fe^3+^ and SO_4_^2−^, Ca^2+^ and F^−^ were first simulated for 40 ns at 298.15 K and 1 atm. Fe^3+^ and SO_4_^2−^ were then introduced, and the production simulation was continued for an additional 40 ns under the same temperature and pressure conditions. Representative sampling points at 5, 10, 20, and 40 ns after the introduction of Fe^3+^ and SO_4_^2−^ were selected from the production trajectory for structural analysis. The Ca–F radial distribution function, g_Ca–F_(r), and coordination number, n_Ca–F_(r), were analyzed at these sampling points. As shown in Figure 8a, both g_Ca–F_(r) and n_Ca–F_(r) progressively increased along the production trajectory, with the maximum g_Ca–F_(r) increasing from 1147.421 at the 5 ns sampling point to 1307.523 at 40 ns and n_Ca–F_(r) increasing from 1.190 to 1.408. These changes reflect the progressive structural rearrangement and association of Ca^2+^ and F^−^ following the introduction of Fe^3+^ and SO_4_^2−^.

As shown in Figure 8b, the F^−^ density profile in the CaF_2_ system exhibits relatively small fluctuations, indicating a comparatively uniform spatial distribution of F^−^. In contrast, the CaF_2_–Fe system shows substantially larger fluctuations in the F^−^ density profile, with pronounced peaks and valleys. The high-density peaks indicate localized regions with greater F^−^ accumulation, whereas the low-density regions reflect a more heterogeneous spatial distribution. These results demonstrate a clear difference between F^−^-enriched and F^−^-depleted regions in the presence of Fe-containing species, providing additional evidence for the enhanced spatial association and aggregation of F^−^ in the CaF_2_–Fe system.

**Figure 9:**
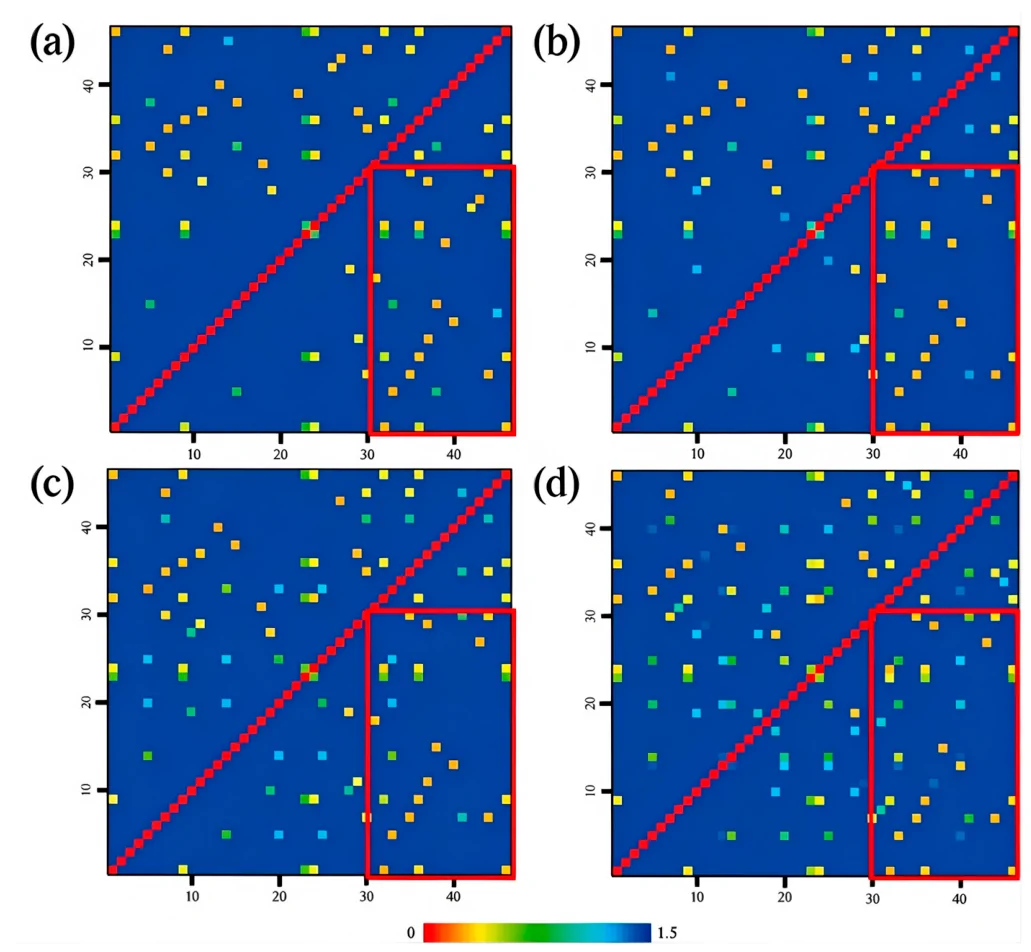
Distance matrix of the minimum distances (nm) between Ca^2+^ and F^−^ with different simulation times in FCS-2. (1–30 are F^−^, 31–45 are Ca^2+^). (**a**) 5 ns; (**b**) 10 ns; (**c**) 20 ns; (**d**) 40 ns.

Figure 9 presents the Ca^2+^–F^−^ distance matrices at representative sampling points of 5, 10, 20, and 40 ns along the production trajectory of the FCS-2 system, as shown in panels (a)–(d), respectively. The indices 1–30 and 31–46 correspond to F^−^ and Ca^2+^, respectively. In the distance matrices, red regions represent shorter Ca^2+^–F^−^ distances, whereas blue regions represent longer distances within the analyzed range of 0–1.5 nm. The regions highlighted by the red boxes show the evolution of close contacts between Ca^2+^ and F^−^. As the production trajectory progressed, the number of close Ca^2+^–F^−^ contacts increased, accompanied by a greater proportion of red regions, indicating progressive structural association and closer spatial contact between Ca^2+^ and F^−^. These observations are consistent with the RDF and coordination-number analyses and provide additional structural evidence for the evolution of Ca^2+^–F^−^ association following the introduction of Fe^3+^ and SO_4_^2−^.

#### 3.4.5. Microscopic Coagulation Behavior

**Figure 10:**
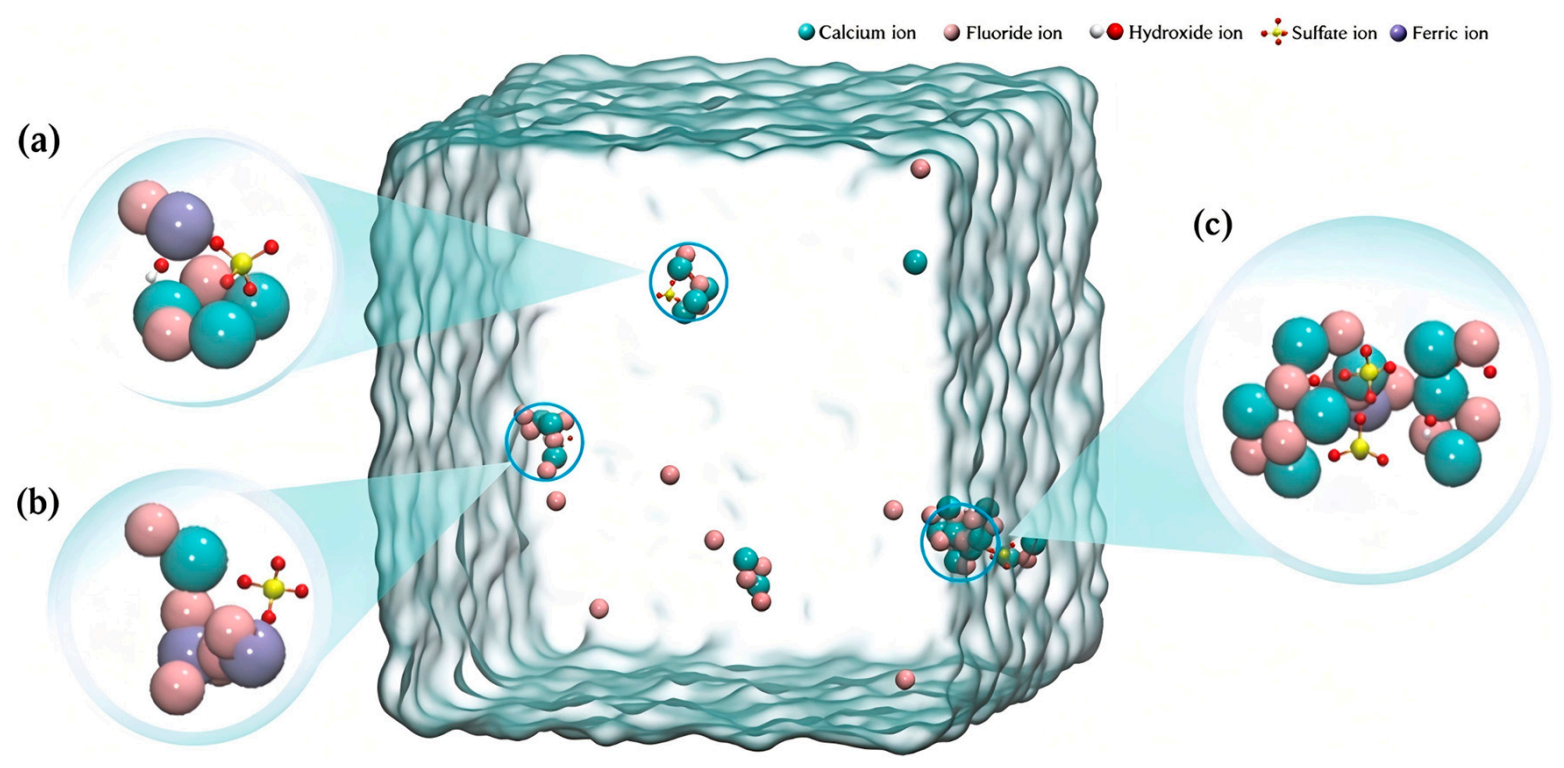
Binding mechanisms of Fe^3+^ and SO_4_^2−^ to other particles during coagulation in FCS-2 system. (**a**) Fe^3+^-Ca^2+^ bridging via OH^−^; (**b**) F^−^-Ca^2+^ coordination mediated by Fe^3+^; (**c**) SO_4_^2−^ bridging between Ca^2+^ and Fe^3+^.

Figure 10 illustrates representative ionic association patterns during the coagulation process. In Figure 10a, Fe-containing species are spatially associated with Ca^2+^ in the presence of OH^−^, suggesting possible OH^−^-mediated interactions between Fe^3+^ and Ca^2+^. In Figure 10b, close association between Fe^3+^ and F^−^ is observed, suggesting that electrostatic interactions involving Fe-containing species may promote the association of F^−^ with Ca^2+^. Figure 10c further shows the formation of a larger multicomponent ionic cluster involving Ca^2+^, F^−^, Fe^3+^, SO_4_^2−^, and OH^−^. Compared with the local ionic associations shown in Figure 10a,b, the simultaneous spatial association of these species indicates the development of a more interconnected ionic structure. Fe-containing species and SO_4_^2−^ may provide additional interaction sites within the Ca^2+^–F^−^ aggregates, while OH^−^ may mediate interactions between Fe^3+^ and Ca^2+^. These cooperative ionic interactions may facilitate the formation of larger CaF_2_-containing aggregates. Previous studies have shown that highly charged cationic species can enhance the charge-neutralization and particle-destabilization ability of PFS [46]. In addition, SO_4_^2−^ may participate in ionic interactions with positively charged species within the Fe-containing clusters, further promoting the formation of interconnected multicomponent aggregates. Overall, these results provide a possible molecular-level explanation for the experimentally observed enhancement of CaF_2_ aggregation and fluoride removal following PFS addition.

## 4. Conclusions

The experimental and characterization results demonstrate that the sequential addition of PFS after CaO precipitation significantly enhances fluoride removal from phosphogypsum leachate through synergistic coagulation and aggregation effects. PFS promotes CaF_2_ precipitate aggregation by modifying particle interactions and facilitating the formation of larger and denser aggregates. The MD results show that pH and CaO/PFS addition sequence significantly influence Ca^2+^–F^−^ association, with pH 12 providing the most favorable condition among the simulated systems and sequential addition exhibiting stronger Ca^2+^–F^−^ association than simultaneous addition based on RDF and coordination-number analyses. The simulations also reveal possible cooperative interactions among Ca^2+^, F^−^, Fe-containing species, SO_4_^2−^, and OH^−^, providing molecular-level insight into the enhanced CaF_2_ aggregation observed experimentally. It should be noted that the MD model simplifies the complex hydrolysis and polymerization behavior of PFS by representing it with selected Fe^3+^ and sulfate ions; therefore, the simulations mainly provide qualitative molecular-level insights into the aggregation mechanism. Future studies involving more comprehensive representations of PFS species are needed to further elucidate the coagulation process.

## Figures and Tables

**Table 1:** Number of individual particles in different systems.

Simulated System	Size (nm)	pH	Ca^2+^	F^−^	Fe^3+^	SO_4_^2−^	OH^−^	H_2_O
FC-1	7 × 7 × 7	pH = 7	15	30	-	-	-	11,170
FC-2	7 × 7 × 7	pH = 12	16	30	-	-	2	11,169
FC-3	7 × 7 × 7	pH = 13	25	30	-	-	20	11,143
FC-4	7 × 7 × 7	pH = 14	115	30	-	-	200	10,811
FCS-1	7 × 7 × 7	pH = 12	16	30	4	6	2	11,148
FCS-2	7 × 7 × 7	pH = 12	16	30	4	6	2	10,733

## Data Availability

Data is contained within the article or Supplementary Material. The original contributions presented in this study are included in the article. Further inquiries can be directed to the corresponding author.

## Supplementary Materials

The following supporting information can be downloaded at: https://www.mdpi.com/article/10.3390/molecules31193524/s1, Figure S1: Evolution of energy, temperature, density, and RMSD during the MD simulation of the FCS-1 system.
