# Supplementary material for: Efficient Removal of Fluoride from Phosphogypsum Leachate: Insights into Microscopic Mechanism of Polyferric Sulfate-Assisted Precipitation via Molecular Simulation
Source: Molecules. 2026 Oct 3;31(19):3524. doi: 10.3390/molecules31193524 (PMC13667218; doi:10.3390/molecules31193524)
Supplement: Supplementary file 1 [file molecules-31-03524-s001.zip › molecules-4524127-supplementary.pdf]

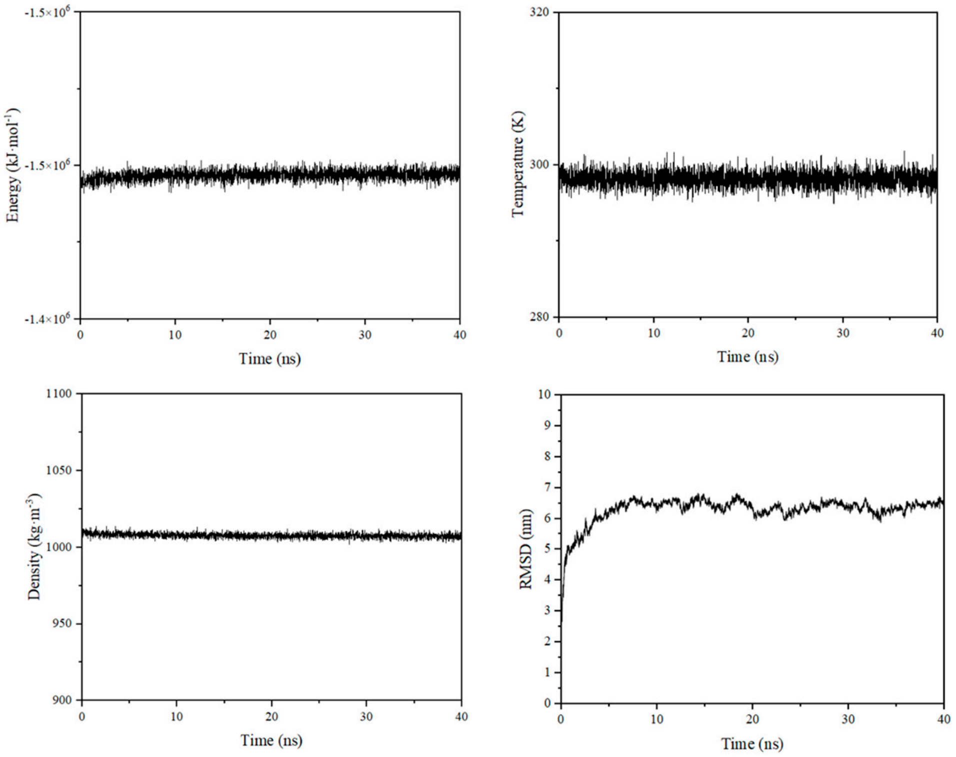

Figure S1. Evolution of energy, temperature, density, and RMSD during the MD simulation of the FCS-1 system
